# Visible Persistence of Single-Transient Random Dot Patterns: Spatial Parameters Affect the Duration of Fading Percepts

**DOI:** 10.1371/journal.pone.0137091

**Published:** 2015-09-08

**Authors:** Maximilian Bruchmann, Kathrin Thaler, Dirk Vorberg

**Affiliations:** 1 Institute of Medical Psychology and Systems Neuroscience, University of Münster, Münster, Germany; 2 Institute for Biomagnetism and Biosignalanalysis, University of Münster, Münster, Germany; 3 Department of Psychology, University of Münster, Münster, Germany; University of Groningen, NETHERLANDS

## Abstract

Visible persistence refers to the continuation of visual perception after the physical termination of a stimulus. We studied an extreme case of visible persistence by presenting two matrices of randomly distributed black and white pixels in succession. On the transition from one matrix to the second, the luminance polarity of all pixels within a disk- or annulus-shaped area reversed, physically creating a single second-order transient signal. This transient signal produces the percept of a disk or an annulus with an abrupt onset and a gradual offset. To study the nature of this fading percept we varied spatial parameters, such as the inner and the outer diameter of annuli (Experiment I) and the radius and eccentricity of disks (Experiment III), and measured the duration of visible persistence by having subjects adjust the synchrony of the onset of a reference stimulus with the onset or the offset of the fading percept. We validated this method by comparing two modalities of the reference stimuli (Experiment I) and by comparing the judgments of fading percepts with the judgments of stimuli that actually fade in luminance contrast (Experiment II). The results show that (i) irrespective of the reference modality, participants are able to precisely judge the on- and the offsets of the fading percepts, (ii) auditory reference stimuli lead to higher visible persistence durations than visual ones, (iii) visible persistence duration increases with the thickness of annuli and the diameter of disks, but decreases with the diameter of annuli, irrespective of stimulus eccentricity. These effects cannot be explained by stimulus energy, which suggests that more complex processing mechanisms are involved. Seemingly contradictory effects of disk and annulus diameter can be unified by assuming an abstract filling-in mechanism that speeds up with the strength of the edge signal and takes more time the larger the stimulus area is.

## General Introduction

Visual illusions vividly demonstrate the possible discrepancy between physical input and phenomenal experience, and often highlight general visual functions by disclosing their malfunctioning under specific conditions. The majority of such illusions affect spatial parameters, e.g. objects look brighter, larger, or more tilted than they actually are. Less common are discrepancies in the temporal domain, probably because they are often more subtle and require dynamic displays. *Visible persistence* is such a discrepancy; it refers to the perceived presence of an object after its physical offset.

A classical definition of visible persistence is that it is one of three components of the broader concept *iconic memory* [1]. Iconic memory in turn is defined as the sensory memory that stores elements of visual information for a very brief period [1,2]; it is also referred to as *visual persistence* [3]. Visual persistence can be further subdivided into *informational*, *visible*, and *neural persistence* [3]. The term iconic memory, as it is typically used, corresponds most to informational persistence, which is described as the knowledge about features of a visual stimulus that remains available after stimulus termination (e.g. information about the location, color or shape). In contrast to informational persistence, visible persistence is defined as the *prolonged percept* of a visual stimulation that continues beyond the physical termination of the stimulus itself [4]. By definition, visible persistence refers to conscious stimulus representations, whereas informational persistence is not necessarily conscious.

Trying to measure the “minimum duration of a perception”, Efron [5,6] observed that the perceived offset of a luminance-defined disk presented for 130 ms or less always occurred about 230 ms after its physical onset. Stimuli presented longer than 130 ms appeared to last for a period that equals their physical duration plus about 100 ms; analogous findings have been obtained in the auditory domain. Thus, stimuli persisted for at least 100 ms and followed–for brief durations–an *inverse-duration effect* (see [3,7] for an overview), which states that visible persistence increases with decreasing stimulus duration. The minimal stimulus duration used by Efron was 5 ms, and produced a visible persistence duration of about 225 ms.

Visible persistence, as studied by Efron and others [3,5–8], cannot be due to retinal afterimages only, as afterimages *increase* with duration. Moreover, visible persistence *decreases* with stimulus intensity (inverse-intensity effect; [4,7]), which, again, is the opposite of what holds for retinal afterimages. Cortical processes are thus likely to contribute to visible persistence, but how, where, and why these processes work is not completely understood, although models exist, which will be discussed later [1,9].

Research on visible persistence has been mainly conducted with two types of stimuli: *first-order* stimuli, i.e. stimuli defined by luminance or wavelength contrasts, have been used by the majority of researchers (see [3,7]), whereas *second-o*rder stimuli, i.e. stimuli defined, e.g., by relative motion have been used by Shioiri and Cavanagh [10].

Both types of stimuli have physically defined on- and offsets. Consequently, they carry three types of signals: *transient onset* signals, *sustained presence* signals and *transient offset* signals. Efron’s studies indicate that sustained signals, i.e., signals that scale with stimulus duration, are not responsible for visible persistence. Di Lollo [11] further showed that iconic memory is triggered at stimulus onset, not at offset. Di Lollo’s task required subjects to report the location of a missing element in successively presented matrices of dots. Theoretically, subjects could have relied on abstract location information (informational persistence; see also [12,13]) rather than a prolonged percept of the dots (visible persistence). However, since the number of persisting dots was much greater than the known capacity limit of informational persistence of 4–5 items [1,2], we can conclude that visible persistence appears to be fundamentally influenced by the onset of a stimulus, and neither by its duration, nor by its offset.

A different line of research has studied persistence of stimuli without an actual offset: in form-from-motion studies [14,15] segments outlining an object are embedded within other segments, which effectively camouflages the object. A relative motion of the embedding segments makes the object appear instantly, whereas stopping the motion results in a percept of the embedded object that gradually fades into camouflage again. Notably, the time course of this type of decaying persistence is in the order of one to two seconds [16–18], at least ten times longer than the visible persistence estimates reported by Coltheart, Di Lollo, or Efron and colleagues. However, the substantial differences between the paradigms make it hard to compare the results directly: classic visible persistence stimuli are defined by luminance rather than by relative motion. In the former case, subjects see a blank display after the stimulus has disappeared, whereas in the latter case the stimulus continues to be present but is camouflaged by other objects. If classic visible persistence is generated at stimulus onset [11], the question arises how to define onset in form-from-motion studies. If the onset of relative motion were defined as the critical onset then no visible persistence could be observed if the motion signal lasts longer than the duration of visible persistence. Demonstrations of form-from-motion clearly show that this is not the case; visible persistence is observed even after very long exposure to relative motion. If the offset of the relative motion triggered persistence of the embedded form, it remains unclear which role relative motion plays for the fading percept after motion-offset. After all, the fading percept typically does not feature motion, neither in the same or the opposite direction of the “inducing” motion.

In order to resolve these uncertainties, we introduce a new type of stimulus, a minor variation of the classical random-dot-kinematogram. Our stimuli consist of two successive matrices randomly filled with black and white pixels. On the transition from the first to the second matrix we flip the luminance polarity of all pixels within a target region that has a simple shape, e.g., a disk. Physically, this corresponds to a single second-order transient signal, approximating a step function per pixel. In contrast to the studies mentioned above, our stimuli therefore feature a single transient onset signal, but neither a sustained signal nor a transient offset signal. Phenomenologically, the stimulus produces the percept of a disk, which sets on abruptly and gradually fades within several tenths of a second. Henceforth, we refer to the physical stimulus as *transient shape*, or simply *transient*, whereas *fading percept* will refer to the consciously perceived, gradually disappearing shape that is generated by the transient (an animated example of a transient shape can be found in the supporting information, S1 Fig). Stimuli as these are common in studies on motion processing (e.g. [19]), but there the stimulus is usually created by repeatedly displacing elements of a pixel matrix from one stimulus frame to the next. Furthermore, subjects are typically asked to detect or identify motion, instead of reporting visible persistence. Stimuli with pixels that change just once have been employed by Sackur [20] for studying metacontrast masking rather than visible persistence; he displaced pixels instead of flipping them. Similar visual stimuli have been employed by Wilson [21,22] for measuring visible persistence in so-called dot bigrams; to construct dot-bigrams, Wilson arranged dots in the shape of letters. These dots were then assigned to one of two subsets that were uninformative with respect to letter identity when presented in isolation. To study persistence generated by onset-signals, the two subsets were successively added to randomly scattered dots and subjects were asked to identify the letter to which the two subsets added up. Wilson measured how the inter-stimulus interval (ISI) between the presentations of the subsets affected identification performance; the analogous procedure was also used with disappearing dots. Wilson [21] reported that on- and offset signals persisted for at least 180 ms and 120 ms, respectively. The transient shapes used here allow us to study visible persistence contaminated neither by sustained signals nor by transient offset signals.

## Experiment I

### Introduction

The purpose of the first experiment was to (i) explore the effects of spatial variations of the stimulus on the duration of visible persistence, (ii) test a “stimulus energy” model of visible persistence which predicts increasing visible persistence duration with number of flipping pixels, and (iii) to cross-validate the judgment-of-synchrony method of estimating the duration of the fading percepts.

To assess the duration of the fading percepts, we adopted the *judgment-of-synchrony* method from Sperling [1] and had subjects adjust the perceived onset of an auditory reference stimulus either to the onset or to the offset time-point of the fading percept. For example, on a given trial a transient shape in the form of an annulus was repeatedly presented together with a tone. The subject was asked to adjust the time-point of the tone onset so that it temporally coincided with the *onset* of the fading percept, yielding a time-stamp of perceived onset. On other trials the subject was to adjust the tone onset such that it temporally coincided with the *termination* or *disappearance* of the fading percept. The transient-shape/reference-stimulus loop was repeated until the subject signals perceived synchrony of the tone’s onset with either the fading percept’s onset or offset. As detailed below, the difference between the perceived onset and offset time-points served as a measure of the duration of visible persistence.

### Methods

#### Subjects

Ten subjects (5 male, 5 female) with normal or corrected-to-normal vision participated in the experiment. They were between 23 and 30 years old (M = 25; SD = 2). All subjects were right-handed; they volunteered for participation and were rewarded with 9 Euro per hour. All procedures were carried out according to the declaration of Helsinki and were approved by the ethical committee of the medical faculty of the University of Münster. In accordance with this approval, all subjects provided written consent to participate in this study.

#### Apparatus and stimuli

All stimuli were presented on a ViewSonic G90fB CRT monitor running at 100 Hz and a resolution of 1024 ✕ 768 pixels at a viewing distance of 60 cm. The mean brightness of the monitor was set to 50 cd/m^2^ (l_min_ = 0.442 cd/m^2^, l_max_ = 100.145 cd/m^2^).

The experiment was run using MATLAB (Version 2010a, The MathWorks) and the Psychophysics Toolbox (Version 3.0.10; [23,24,25]).

**Fig 1:**
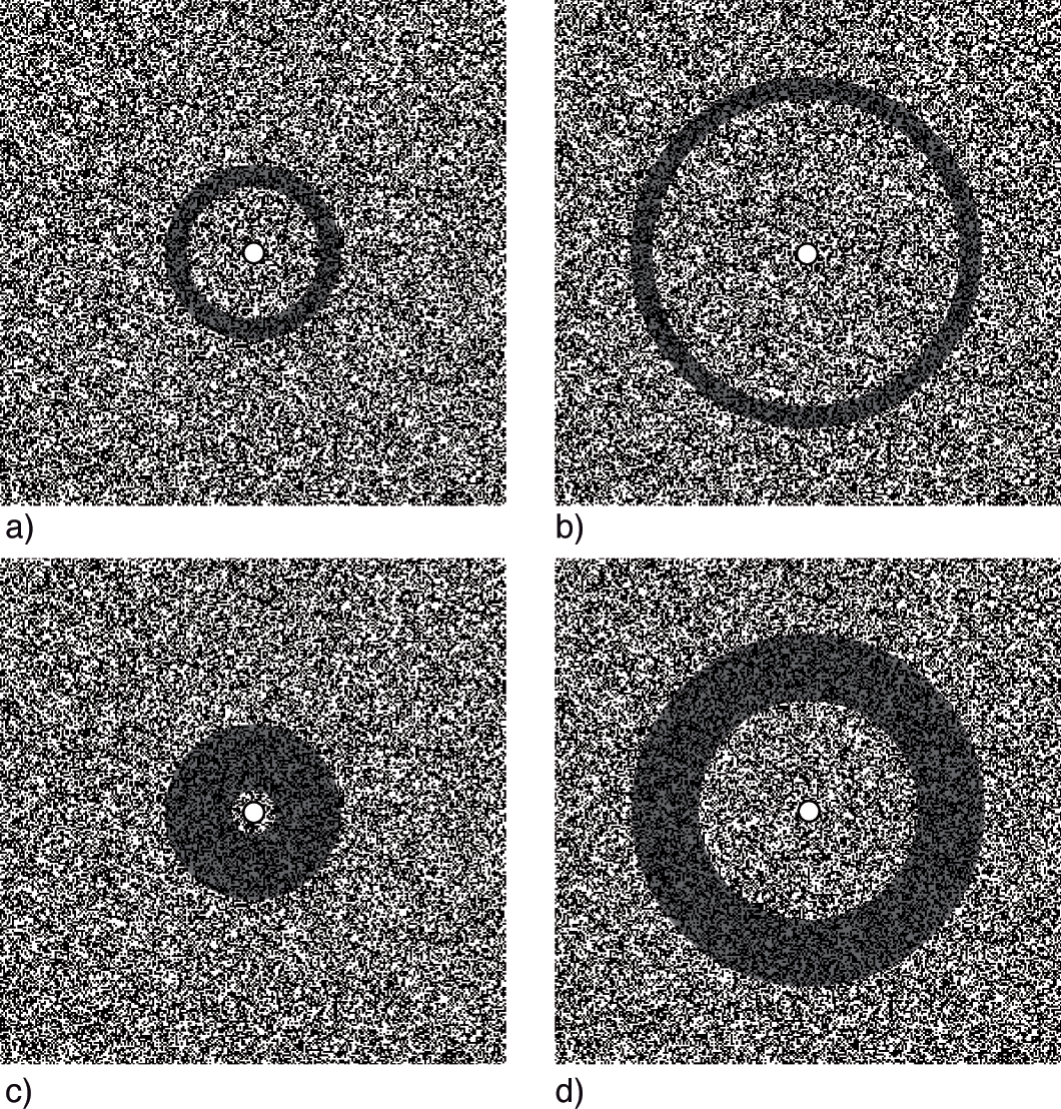
Illustration of the four different annuli, drawn to scale with the central 300 ✕ 300 pixels of a 600 ✕ 600 pixels random dot matrix. The shaded regions illustrate the area where pixels reversed luminance polarity. The actual experiment featured only the random-dot matrix and the central fixation mark.

The random-dot matrices consisted of 600 ✕ 600 pixels, randomly black or white. The total matrix subtended 19.15 ✕ 19.15 degree of visual angle (deg) and was positioned in the center of the screen. A trial consisted of the alternating presentation of two matrices which was repeated every 1.5 to 5 s until the subject gave a specific response (see Procedure). The two matrices were identical except for an annulus shaped region in the center of the matrix. The transition between the matrices triggered the fading percept. The annuli had outer diameters of either 100 or 200 pixels (3.19 deg or 6.39 deg), henceforth referred to as *small* and *large* (throughout this report, *Size* will refer to stimulus diameter), with thickness either 25 or 75 pixels (0.80 deg or 2.89 deg), respectively, referred to as *thin* and *thick*. Fig 1 depicts the central 300 ✕ 300 pixels of a random-dot matrix with shaded regions indicating the four different annuli. The stimulus dimensions were chosen to create differently proportioned annuli with identical stimulus energy: the surface area of small, thick annuli was identical to that of large, thin annuli, thus the same amount of pixels changed within the same time.

The task was to adjust the onset time-point of a reference stimulus to the on- or offset time-point of the fading percept (see Procedure). As reference stimuli we used either a 1000 Hz sine tone with 500 ms duration of (with 5 ms logistic on- and off-ramps) or two black disks with a diameter of 2.55 deg presented simultaneously at the vertical center left and right next to the matrices (displaced 12.77 deg left and right from the center).

### Procedure

**Fig 2:**
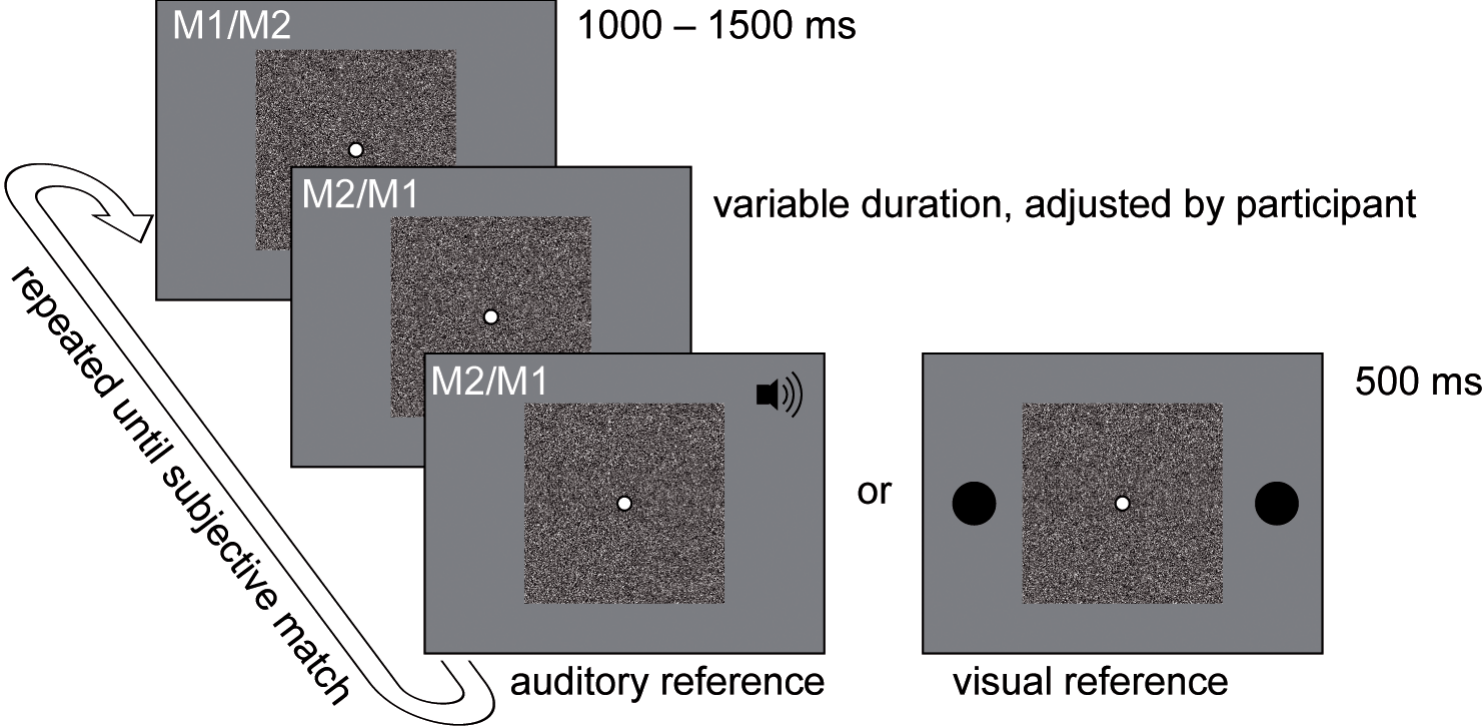
Illustration of a trial sequence: a randomly generated matrix (M1) was presented for a randomized duration between 1000 and 1500 ms. Then, this matrix was replaced by a second matrix (M2), which was identical to M1 except that all pixels in a annulus shaped region reversed luminance polarity. After a variable duration, either an auditory or a visual reference stimulus was presented for 500 ms, while M2 remained on the screen. The sequence was repeated, now with M2 as the first and M1 as the second matrix. Participants could adjust the interval between the matrix transition and the reference stimulus onset using four keys on an external response box in steps of -100, -10, 10, and 100 ms, respectively. The sequence was repeated until the participant indicated with a fifth key that perceptual synchrony between the onset of the reference stimulus and–depending on the task in a given trial–the on- or offset of the fading percept was established.

Each experimental trial consisted of at least two repetitions of the same stimulus sequence (see Fig 2): a random-dot matrix (M1; randomly generated at the beginning of each new trial) was presented at the center of the screen. The subjects were to place the head in a chin-and-forehead rest and fixate the center of the screen. Subjects were to focus on a white dot with a black contour (diameter ø = 0.32 deg) in the center of the matrix. After a randomized interval between 1000 and 1500 ms the matrix was replaced by M2. Matrices M1 and M2 were identical except for the annulus shaped target region, where all pixels reversed luminance polarity. This second-order transient shape triggered the fading percept. An example of such a stimulus was presented during the on-screen instructions; the subjects were asked to verbalize their percept. All subjects reported to experience fading percepts as described above.

As noted above, Sperling’s judgment-of-synchrony-method was used to measure visible persistence, defined as the duration of the fading percept. We provided subjects with as much time as they needed for matching the onset or the offset of the fading percept with the onset of the reference stimulus in each trial (which was repeated in a loop until the subject was satisfied with the adjustment). Adjustments were made by pressing one of five keys on an external response box, which changed the onset time point of the reference stimulus in the following way: Some time after the M1-M2-transition at *t*_*T*_ the auditory or visual reference stimulus appeared (Δ*t* = *t*_*R(on)*_ *−t*_*T*_). Any key press during the whole sequence was recorded. The keys 1 to 4 changed Δ*t* by -100, -10, 10, or 100 ms, respectively. Because of the display refresh rate the minimal increment (or decrement) was limited to 10 ms for the visual reference stimulus. To compare the subjects’ accuracy between reference modalities, we used the same increment and decrement sizes for the auditory reference stimulus as well. By pressing a fifth key, subjects indicated that a temporal match between the fading percept and the reference stimulus had been established. The fifth key also initiated the next trial.

**Fig 3:**
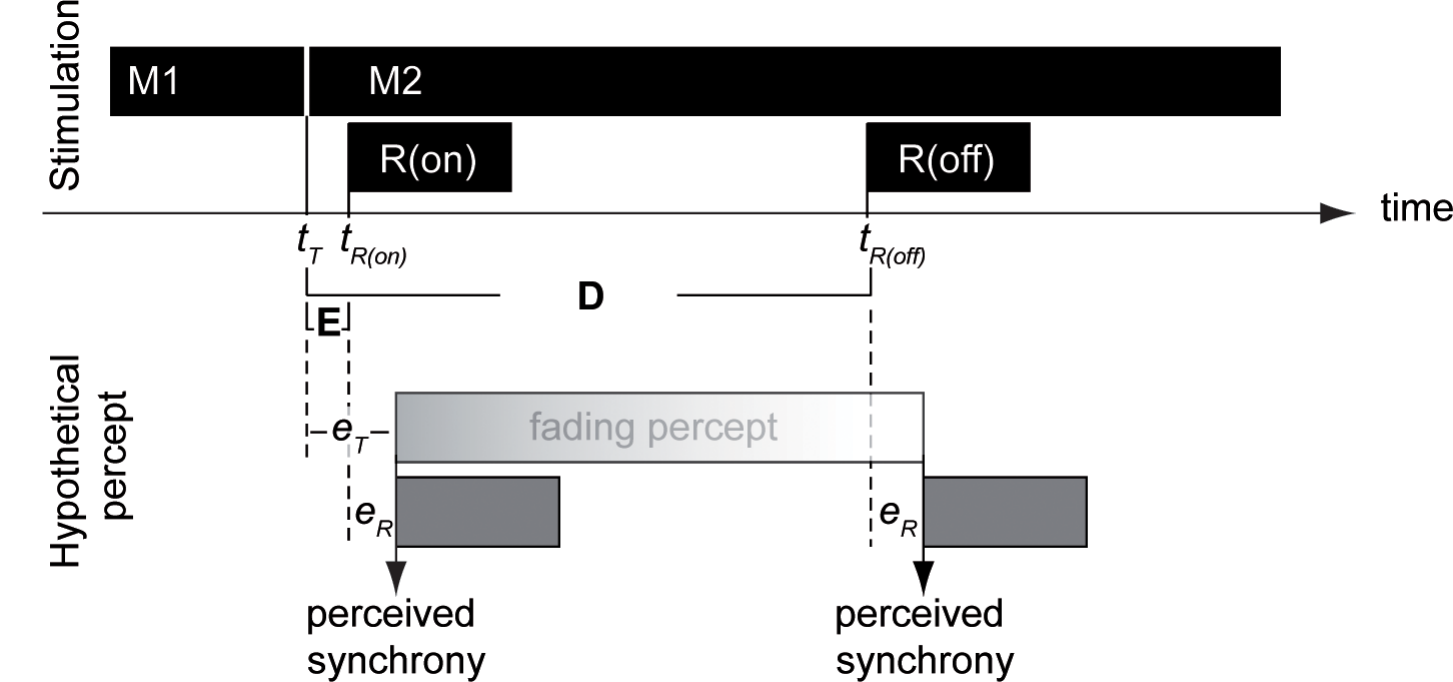
Rationale underlying the judgment-of-synchrony method (adopted from Sperling, 1967): the upper part shows the physical stimulation. M1 and M2 refer to the two random dot matrices; *t*_*T*_ refers to the time point of transition. *R(on)* and *R(off)* are the reference stimuli presented at *t*_*R(on)*_ and *t*_*R(off)*_, respectively. In the experiment, trials contained either *R(on)* or R(off), but not both. The method admits unknown delays between the physical onset of a stimulus and the onset of its percept. This is denoted by *e*_*T*_ (i.e. the delay between the matrix transition and the onset of the fading percept) and *e*_*R*_ (i.e. the delay between the onset of the reference stimulus and its percept). Note that despite these unknown delays the duration of the percept of the transient shapes (i.e. *VP*) can be deduced from the time points of the matrix transition and the reference stimulus onsets: *VP = D–E*.

*On-trials* required subjects to adjust Δ*t* so that the perceived onset of the reference stimulus coincided with the perceived onset of the fading percept. On *off-trials* subjects were to adjust Δ*t* so that the perceived onset of the reference stimulus coincided with the perceived offset of the fading percept. Following the notation of Efron [5], *E* and *D* denote the values of Δ*t* at which onset-onset synchrony and onset-offset synchrony are established. Since the phenomenological offset was gradual, subjects were instructed to judge when the random-dot matrix appeared as a homogenously textured background. Fig 3 illustrates the rationale.

The levels of the experimental factors *Size*, *Thickness*, and *Reference Modality* were chosen randomly on each trial. After every ten trials the *Task* alternated between onset and offset judgment; the first task in the experiment was always an onset judgment. The German words corresponding to ‘onset’ or ‘offset’ (‘ERSCHEINEN’, ‘VERSCHWINDEN’) were continuously presented below the noise matrix to remind the subjects of the current experimental task.

The initial step size Δ*t* on each trial was chosen as follows: if the experimental condition was shown to a given participant for the first time, Δ*t* was set to ε, where ε was chosen randomly from the set [-30, -20, -10, 0, 10, 20, or 30 ms]. If it was not shown for the first time, Δ*t* was set to $\bar{E}+\varepsilon$, or $\bar{D}+\varepsilon$ on on- or off-trials, respectively, where $\bar{E}$ and $\bar{D}$ refer to the average estimate of all preceding trials of that condition. This semi-adaptive procedure was used to reduce the number of stimulus sequences needed for narrowing down on perceived synchrony. The random jitter ε was introduced to discourage rash confirmations.

Each experimental condition was repeated 20 times, resulting in 320 trials. The measurements were distributed across two to four sessions of 0 to 90 min each, separated by one to four days. Each session started with four practice trials, two on- and two off-trials. The complete experiment required between 105 and 237 min (M = 162 min).

### Results

The data indicated that *D *(i.e. the time-stamp of perceived onset-offset synchrony) and *E* (i.e. the time-stamp of perceived onset-onset synchrony) remained nearly constant from the beginning to the end of the experiment. One subject, however, diverged from this pattern by setting very low values for *D* at the beginning of the experiment, which then increased monotonically. We interpreted this as a misunderstanding of the experimental task and excluded this subject’s data from the analysis, leaving data of nine subjects included in the analyses.

**Fig 4:**
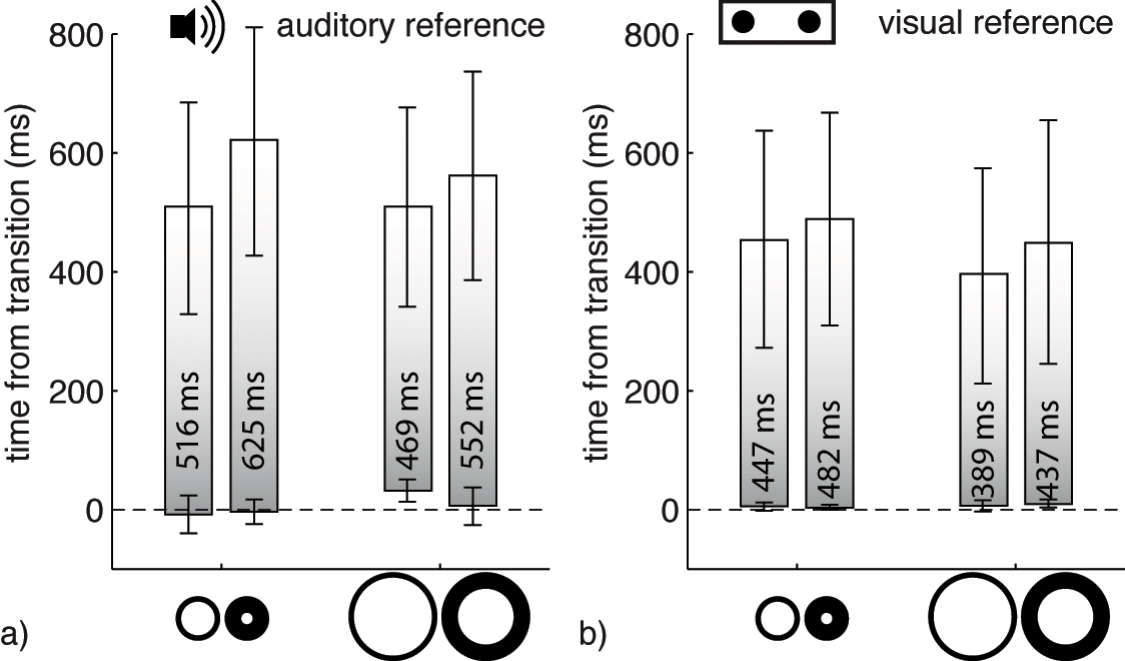
Mean onset and offset delays, E and D, respectively. The lower end of each bar represents E, i.e. the delay between the physical onset of the reference stimulus and the physical onset of the transient shape, at which both onsets were perceived as simultaneous. The upper end of each bar represents D, i.e. the delay between the physical onset of the reference stimulus and the physical onset of the transient shape, at which the onset of the reference stimulus and the offset of the transient shape were perceived as simultaneous. Following the rational illustrated in Fig 3, the length of each bar indicates *VP*, i.e. the mean durations of the fading percept, measured by auditory (a) and visual (b) reference stimuli. The average difference between E and D (i.e. *VP*) is depicted on each bar. Annulus symbols illustrate the small / large and thin / thick conditions, respectively. Error bars indicate 95% confidence intervals of the interindividual means.

Two separate 2 ✕ 2 ✕ 2 (*Size*, *Thickness*, and *Reference Modality*) ANOVA for repeated measurements were conducted, with *E* and visible persistence (*VP)* as dependent variables, respectively. *E-*values were analyzed first to check whether the time of perceived onset was influenced by the experimental factors. As Fig 4 indicates, this was not the case (all p > 0.1).

The analysis of *VP* yielded a significant main effect of *Modality*, F(1,8) = 13.15, p = .007, showing that auditory reference stimuli lead to longer visible persistence duration estimates than visual reference stimuli (*VP*_*aud*_ = 540.8 ms; *VP*_*vis*_ = 438.6 ms). A main effect of *Size*, F(1,8) = 28.03, p < .001, showed that small annuli persisted longer than large ones (*VP*_*small*_ = 517.5 ms; *VP*_*large*_ = 461.9 ms). Finally, thick stimuli persisted longer than thin stimuli (*VP*_*thick*_ = 524.2 ms; *VP*_*thin*_ = 455.2 ms), as shown by a significant main effect of *Thickness*, F(1,8) = 39.39, p < .001. None of the two-way and three-way interactions was significant. Closest to significance was the interaction of *Thickness *✕ *Reference Modality*, F(1,8) = 3.59, p = 0.09, with the thickness-difference appearing to be slightly larger in the auditory compared to the visual condition (*VP*_*thick/aud*_−*VP*_*thin/aud*_ = 96.2 ms; *VP*_*thick/vis*_−*VP*_*thin/vis*_ = 41.7 ms). All other interactions were far from significance (all p > .311).

As the error bars in Fig 4 indicate, onsets were perceived more precisely than offsets. To test this, we performed a 2 ✕ 2 ✕ 2 ✕ 2 (*Size*, *Thickness*, *Reference Modality*, *Onset vs*. *Offset*) repeated measures ANOVA of the standard deviations (SD) of the adjustments. The factor *Onset vs*. *Offset* turned out to be highly significant, F(1,8) = 26.17, p < .001, with SDs of offset judgments being about twice the SDs of onset judgments (*SD*_*Onset*_ = 26.9 ms; *SD*_*Offset*_ = 45 ms). Additionally, the 2-way-interaction *Reference Modality* ✕ *Onset vs*. *Offset* was significant, F(1,8) = 9.94, p < 0.05, indicating that the Onset/Offset SD-difference was stronger in the visual than in the auditory modality. The significant 3-way-interaction *Reference Modality* ✕ *Size* ✕ *Onset vs*. *Offset* suggests that, with auditory reference stimuli, the Onset/Offset SD-difference is nearly absent with small annuli but present with large annuli, whereas with visual reference stimuli the differences are about equally large, F(1,8) = 5.91, p < 0.05.

### Discussion

On average, the fading percepts persisted for half a second. Phenomenologically, the percepts arose abruptly and disappeared gradually. This observation is backed by the larger standard deviations of offset-judgments than of onset judgments. Because of the gradual decay the fading percept may remind one of classical aftereffects, like retinal afterimages, or successive contrast effects. In contrast to these effects, however, visible persistence of transient shapes does not appear to be a simple function of stimulus energy: small / thick annuli and large / thin annuli shared the same number of luminance-reversing pixels and were thus identical in total stimulus energy. Yet, of all experimental conditions, these two differed most, calling for explanations of visible persistence that are not based just on stimulus energy. (Note: stimulus energy here refers to the distal rather than to the proximal stimulus, i.e. the retinal image. To make precise claims about proximal stimulus energy optical aberrations by the cornea, the optical media and the retina would have to be taken into account. However, the size of the difference in visible persistence between small / thick and large / thing annuli makes it extremely unlikely that the two stimuli would trigger equally long-lasting percepts if equated for proximal stimulus energy.)

Despite very large inter-individual variability (SD of *VP* across subjects: 383.9 ms) the intra-individual effects of spatial manipulations were extremely consistent across subjects. Thick annuli persisted about 70 ms longer than thin ones, and small annuli persisted about 60 ms shorter than large ones. On the one hand, the consistency of effects motivates conclusions about the role of thickness and size for the persistence of annuli. The positive effect of thickness and the negative effect of size might be explained by a mechanism akin to brightness filling-in [26–28], where the representation of surfaces is the result of a diffusion-like spread of activation generated at the boundaries of a stimulus. According to this hypothesis, thicker stimuli feature a longer filling-in distance, which might prolong the process, whereas stronger edge signals (due to size increases) trigger faster filling-in, which might shorten the process. We will elaborate on this idea further in the General Discussion. On the other hand, the spatial manipulations in Experiment I were chosen in an exploratory fashion. Varying size and thickness of annuli necessarily confounds other variables, such as surface area and eccentricity. Eccentricity might be critical because it affects the temporal response properties of the visual system via the distribution of M- and P-cells on the retina, featuring more fast-responding M-cells in the periphery and more sustained responding P-cells near the fovea [29,30]. The temporal integration model by Groner, Bischof, and Di Lollo [31] is a computational model for visible persistence that is based on the response properties of sustained cells. A greater proportion of sustained relative to transient cells activated by smaller, hence more centrally presented annuli could thus explain the observed size effect. Experiment II will therefore be aimed at de-confounding the size of the stimuli and their eccentricity by varying the size of stimuli and their position in the visual field independently.

Of which importance is the modality of the reference stimuli? We found longer visible persistence estimates for auditory compared to visual reference stimuli. Reference modality, however, did not have an effect on the time of perceived onset (*E)*. Thus, differences between the modalities cannot be explained by a relative processing delay of one modality over the other. One explanation could be that the peripheral visual stimuli triggered eye movements, which instantly made the fading percepts disappear. We assume that any obvious deviation from the typically perceived gradual decay, such as an abrupt disappearance following an eye movement, would not distort the synchrony estimates, as an observer would presumably not confirm his or her estimate in case of such an atypical percept, but would instead let the trial repetition continue. However, small and unnoticed eye movements around the onset of the reference stimulus may have caused some bias towards shorter visible persistence estimates. Also in favor of auditory over visual reference stimuli is the improved accuracy of judgments, as evidenced by much smaller standard deviations.

Until now the validity of the judgment-of-synchrony-method has been taken for granted. However, duration judgments of gradually fading stimuli, or in the present case, gradually fading *percepts* without a correspondingly decaying stimulation might be affected by other factors than the actual stimulus (or percept) duration. Therefore, in Experiment II we sought to validate the method by measuring the relation between judgment-of-synchrony-derived durations and the durations of stimuli that actually decay physically smoothly in time.

## Experiment II

### Background

In the past heated debates have been conducted over what exactly is measured in experiments on visible persistence [32–34]. In Experiment I, we assumed that the judgment-of-synchrony-method provides valid measures of the duration of conscious percepts, rather than of the period during which abstract stimulus information is available. The fading percepts triggered by the second-order transients are characterized by their sharp onset and their gradual decay. Thus, the validity of subjective duration estimates via the judgment-of-synchrony-method might be questioned as there is no direct way of assessing the psychophysical relation between perceived and physical duration (which is zero constantly). To tackle this problem, we created dynamic visual stimuli that physically decay in time and can thus serve as a proxy for the fading percepts. We tried to mimic their perceptual appearance as closely as possible by manipulating the contrast between bright and dark pixels in the target region as a function of time and had our subjects assess either the perceived onset or the offset of this contrast modulation on separate trials. Demonstrations of this stimulus are provided as supporting information (S2 Fig) to this article.

### Methods

#### Subjects

Four experienced subjects (authors KT and MB; IS and SM were unaware of the purpose of the research) took part in the validation experiment. All procedures were carried out according to the declaration of Helsinki and were approved by the ethical committee of the medical faculty of the University of Münster. In accordance with this approval, all subjects provided written consent to participate in this study.

#### Apparatus and stimuli

The same apparatus as in Experiment I was used, except that the subjects changed Δt no longer by means of four keys but by turning a control knob (PowerMate by Griffin Technology Inc.). The reference stimulus was the same 1000 Hz sine tone with 500 ms duration as in Experiment I.

The amount by which the control knob was turned corresponded linearly to the change in Δt on the consecutive sequence repetition. Turning the knob by 90° to the left or right corresponded to changes in Δ*t* by about –1000 and 1000 ms, respectively. Subjects confirmed a trial by pressing the control knob down. The random-dot matrices had the same dimensions as in Experiment I (600 ✕ 600 pixels, corresponding to 19.15 ✕ 19.15 deg). Instead of transient shapes we presented gray discs with a radius of 1.75 deg, centrally embedded in the random-dot matrix and varied their transparency over time, which is technically identical to changing the luminance contrast of the bright and dark pixels in the target region. The average brightness of the target region was always identical to that of the surrounding part of the matrix.

**Fig 5:**
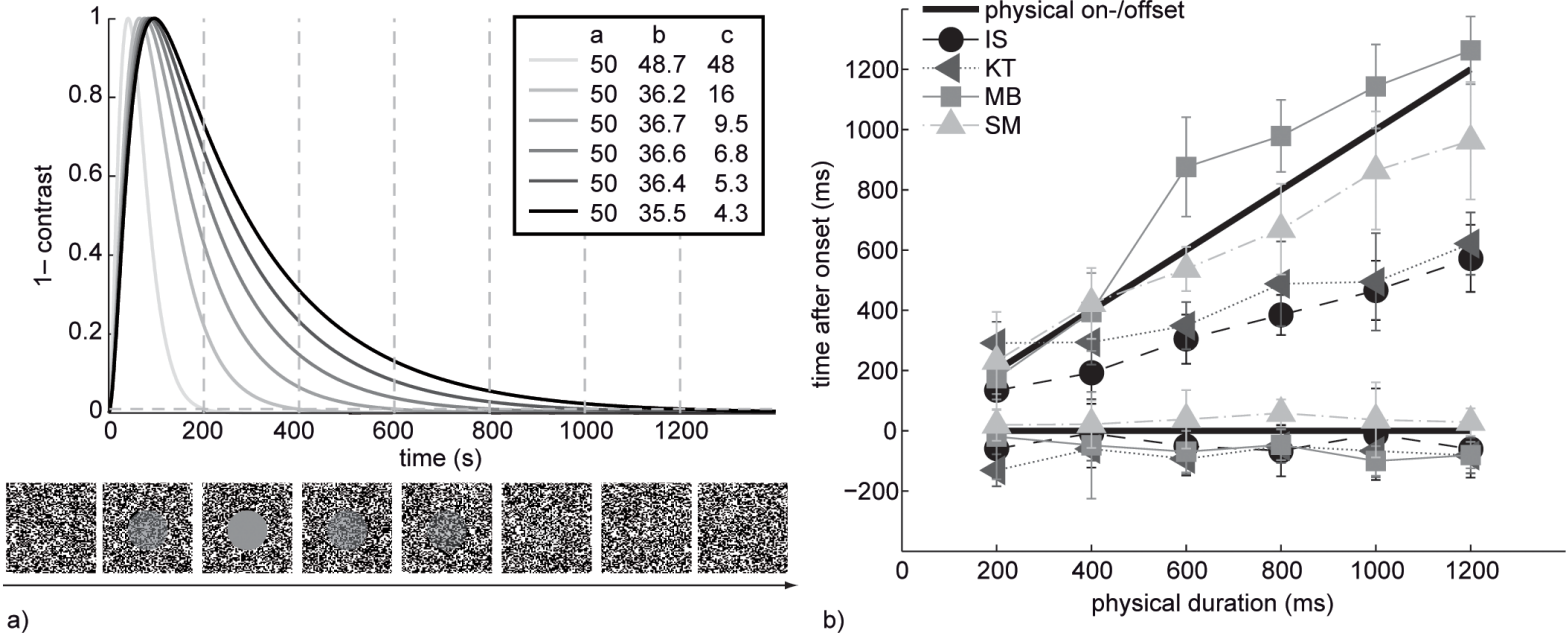
(a) Contrast variation in the target region as a function of time. Six functions were generated by keeping *a* constant and choosing *b* and *c* such that *f(t)* = 0.01 at *t* = 200, 400, 600, 800, 1000, or 1200 ms, as illustrated by gray dashed lines. The sequence of random dot stimuli below the graph illustrates the contrast dynamics in the stimulus area over time (pixel size or background pattern size are not drawn to scale). A contrast reduction of 1 corresponds to a homogenous grey disk, equal to the average luminance of the black and the white pixels. (b) Onset and offset adjustments (means and SEs) by four observers. The solid straight lines indicate the stimulus on- and offsets, respectively, (where offset is defined as *f(t)* = 0.01). For all subjects, perceived onset was independent of physical duration, whereas perceived offset linearly increased with physical duration.

For the temporal dynamics of stimulus contrast, we chose the impulse response function of a three-stage linear system defined by

$$f(t)=D[(c-b)e^{-at}\;+\;(b-a)e^{-ct}\;-\;(c-a)e^{-bt}],$$

where *a*, *b* and *c* are decay parameters of three sequential visual stages, and D = D(a, b, c) is a norming constant such that the peak of the function equals 1. This form of the postulated persistence function was chosen mainly for its flexibility and its intuitive plausibility, but note that *f(t)* can be seen as a generalization of the temporal integration model proposed by Groner et al. [31]. The 3-stage extension was chosen in order to provide more flexibility in mimicking the decay time-course of the fading percepts. We kept *a* constant at *a* = 50 while varying *b* and c such that *f(t*_*i*_ *)* = 0.0 at *t*_*i*_ = 200, 400, 600, 800, 1000, or 1200 ms. The resulting decay functions are shown in Fig 5(A).

### Procedure

The procedure closely followed that of Experiment I. Each experimental trial consisted of at least two repetitions of the same stimulus sequence: a random-dot matrix was presented for a random duration between 1000 and 1500 ms, together with the same central fixation dot as described above. The matrices were generated randomly on each single sequence repetition. Below the matrix, the German words for onset or offset (‘ERSCHEINEN’, ‘VERSCHWINDEN’) indicated the task on a given trial. The order of onset and offset trials was random. After the fixation interval, the proxy stimulus was presented with one of the six possible time-courses, also chosen randomly. On the first repetition of a given trial, the onset of the reference tone was set to a random delay (Δ*t)* between -200 and 200 ms relative to the onset or the offset (depending on the current task) of the proxy stimulus. For all following repetitions Δ*t* was held constant at the value that the subject adjusted by means of the control knob. The stimulus sequence was repeated until the subjects indicated by pressing the control knob that he or she had established perceptual synchrony between the on- or the offset of the proxy stimulus and the onset of the reference tone. Each of the twelve experimental conditions (six durations, on- or offset task) was repeated ten times. The order of the 120 trials was randomized for each subject.

### Results

No subject showed any systematic dependence of perceived onset time on physical duration, as can be seen in Fig 5(B). For each subject we calculated separate two-way ANOVAs with factors *Duration* (six levels) and *Task* (on- or offset) on the difference between estimated time point and physical time point. The two-way interaction was significant in all cases, all F(5,119) > 11.97, all p < .001, indicating that, as expected, onset and offset judgment were differently affected by stimulus duration. To break down the interaction we calculated two separate one-way ANOVAs with factor *Duration*. The first ANOVA dealt with the onset, the second with the offset. The constant term of the ANOVA revealed that three out of four subjects set the onset of the reference tone slightly before the onset of the target stimulus to perceive synchrony (IS: F(1,9) = 7.84, p = 0.021; KT: F(1,9) = 56.58, p < .001; MB: F(1,9) = 27.62, p < .001), whereas SM set the onset of the reference tone slightly after the onset of the target stimulus, F(1,9) = 9.73, p = .012. For subject KT the perceived onset time also appeared to slightly depend on stimulus duration, as reflected by a marginally significant main effect of *Duration*, F(5,45) = 2.97, p = 0.057 (after Greenhouse-Geisser correction due to violation of the sphericity assumption).

A second ANOVA on estimated offset time points revealed a main effect of duration, all F(5,45) > 17.02, all p < .001. More importantly, in all subjects the estimates followed a linear trend, all F(1,9) > 75.08, all p < .001, thus revealing a monotonous increase in perceived offset time with increasing physical duration.

The standard deviations of on- and offset judgments were as follows: IS: *sd*_*on*_ = 107.01 ms, *sd*_*off*_ = 92.73; KT: *sd*_*on*_ = 57.49 ms, *sd*_*off*_ = 104.83; MB: *sd*_*on*_ = 88.74 ms, *sd*_*off*_ = 122.78; SM: *sd*_*on*_ = 73.90 ms, *sd*_*off*_ = 149.24. Thus, for three out of four subjects, the onset was estimated more precisely than the offset. For subject IS offset estimates were slightly more precise than onset estimates. Notably, IS was also the subject with the greatest underestimation of the offset time point, which could indicate that IS used a comparably high threshold for offset judgments, where the decay function is much steeper.

### Discussion

The results show that the judgment-of-synchrony-method provides valid estimates of the perceived duration of smoothly decaying stimuli: increasing physical duration resulted in an almost linear increase of the judgment-of-synchrony-estimates. Moreover, the data again suggest different offset criteria as the likely source of the high inter-individual variability observed in Experiment I. Note that although the same stimuli were used for all subjects, one subject indicated the perceptual offset of the longest-lasting stimulus at about 500 ms, whereas another subject set the reference stimulus at about 1200 ms. Despite these large inter-individual differences, the data are qualitatively consistent: for each subject, they reveal an approximately linear relationship to physical stimulus duration. Additional support for the assumption that the inter-individual differences in perceived duration reflect individually different offset criteria can be seen in the fact that the subject with the highest offset-criterion (IS) showed the smallest standard deviation across trial repetitions in offset estimates. We see this as a direct consequence of the increase in the steepness of decay function with an increase in criterion level.

We conclude that the judgment-of-synchrony-method yields valid estimates of perceived duration when applied to gradually fading stimuli. However, this interpretation rests on the assumption that smoothly decaying contrast stimuli are comparable to transient shapes. However, the properties of the transient-shape percept cannot be measured directly, and consolidated computational models for the underlying processes do not exist; thus, phenomenological comparisons only support this interpretation. We invite the reader to check on the subjective comparability of transient shapes and the decaying proxy stimulus by viewing the demonstrations to be found as supporting information to this article (S2 Fig).

## Experiment III

### Introduction

The key findings of Experiment I were an increase of visible persistence with annulus thickness but a decrease with annulus size. As these two factors confound stimulus size and retinal location, i.e. eccentricity, we designed Experiment III to dissociate effects of stimulus size and eccentricity. We expected that, in general, larger transient shapes persist longer than smaller ones. This prediction was based on two findings on visible persistence for gratings: visible persistence increases with the number of cycles in a grating [35,36] and the area that the grating subtends [37]. These two reports seem to contradict each other regarding the question whether visible persistence increases with spatial frequency or with the number of cycles; however, both lead to the same prediction for transient random-dot stimuli: For constant pixel size, larger transient shapes cover larger areas and thus more pixels; therefore, we expect that visible persistence increases with stimulus size.

Moreover, we predicted negative effects of eccentricity on visible persistence, as the ratio of quick and transient-responding M-cells to delayed and sustained-responding P-cells increases with eccentricity [30], and because visible persistence has been related to the output of sustained channels in the temporal integration model [31]. Taken together, these two effects could explain why larger (i.e. more eccentric) annuli persisted less than smaller ones, and why thicker (i.e. covering more area) annuli persisted longer than thinner ones.

### Methods

#### Subjects

Nine Subjects (4 male, 5 female; two of which had also participated in Experiment I), with normal or corrected-to-normal vision participated in Experiment III. They were between 23 and 31 years old (M = 26.56; SD = 3.39). All subjects were right-handed; they volunteered for participation and were paid 9 Euro per hour. All procedures were carried out according to the declaration of Helsinki and were approved by the ethical committee of the medical faculty of the University of Münster.

#### Apparatus and stimuli

The same stimulus set-up as in Experiment I and II was used. As in Experiment II subjects changed Δ*t* by turning the control knob (PowerMate by Griffin Technology Inc.). The amount by which the control knob was turned was proportional to the change in Δt. Turning the knob by 90° to counter-clockwise (clockwise) corresponded to changes in Δ*t* by about –1000 (1000 ms). This change became effective on the next sequence repetition. Subjects finished the sequence repetition by pressing the control knob which indicated that perceptual synchrony was achieved. Details were identical to those of Experiment 1, except for the following:

Disks rather than annuli were used as transient shapes, with diameters of 1.5, 3, 4.5, or 6 deg (factor *Size*). The center of each disk was displaced from the center of the matrix by either 3, 4.5, 6, or 7.5 deg (factor *Eccentricity*), in a random direction.

The same auditory reference stimulus as in Experiment I and II was used throughout.

### Procedure

As before, subjects were instructed to maintain gaze on the central fixation mark, even though stimuli would appear in the visual periphery. The experiment started with four practice trials, two on- and two off-trials, i.e. two trials on which the onset of the reference stimulus was to be matched to the onset of the transient shapes, and two trials on which the onset of the reference stimulus was to be matched to their offset.

As in Experiment I a trial consisted of a stimulus sequence that was repeated until subjects had established perceptual synchrony of the reference tone onset either with the onset or with the offset of the fading percept. On- and off-trials were presented in blocks of 10 trials as in Experiment I. The number of trials per condition was reduced from 20 to 10. Also different from Experiment I, the location of the transient shape randomly varied while eccentricity remained fixed.

### Results

As in the previous experiments we first checked whether the onset of the fading percept (*E*) was affected by the experimental parameters, here the size and the eccentricity of the transient shapes. We hypothesized that these parameters would not affect *E*. Second, we analyzed the effect of size and eccentricity on *VP* (the difference between perceived off- and onset time). We hypothesized an increase in *VP* with size and a decrease in *VP* with eccentricity. Two separate 4 ✕ 4 (*Size* ✕ *Eccentricity*) repeated measures ANOVAs for were conducted, with *E* (perceived onset time) and *VP* as dependent variables. Greenhouse-Geisser correction was used when the sphericity assumption was violated; corrected p-values are indicated by asterisk (*).

**Fig 6:**
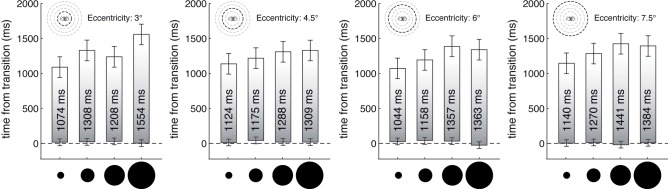
Judged duration of fading percepts, as a function of stimulus size and eccentricity. Bars indicate the duration of the fading percept of the transient shapes, measured by auditory reference stimuli. Bar lengths show the average persistence duration (i.e., *VP* = *E*—*D)* for different disk sizes (1.5, 3, 4.5, or 6 deg in diameter). Each plot corresponds to one eccentricity level (3, 4.5, 6, or 7.5 deg). Pictograms for disk size and eccentricity are drawn to scale. Error bars indicate the 95% confidence for the within-subject effect *Size* (Loftus and Mason, 1994).

As Fig 6 indicates, the factors did not affect *E*, the time of perceived onset (all p > 0.46).

The analysis of *VP* yielded a significant main effect of *Size*, F(3,24) = 13.74, p < .002*, no effect of eccentricity (p = .61), and no interaction of *Size* × *Eccentricity* (p = .164*). Averaged over eccentricities, *VP* linearly increased with disk size, *VP* = 67.78 * *size*(*deg*) + 1007.95, F(1,8) = 16.773, p = .007.

The analysis of the standard deviations by a 4 ✕ 4 ✕ 2 (*Size*, *Eccentricity*, *Onset vs*. *Offset*) ANOVA for repeated measurements yielded a significant main effect of *Onset vs*. *Offset*, F(1,8) = 16.7, p < 0.01. The SDs show that onset judgments were more than twice as precise as offset judgments (*SD*_*Onset*_ = 171.9 ms; *SD*_*Offset*_ = 442.8 ms).

### Discussion

Stimulus size, defined as disk radius, systematically affected the duration of visible persistence, prolonging it on average by 68 ms per degree of radius. Notably, visible persistence was linearly related to radius rather than to stimulus area, or stimulus energy. This relationship was unaffected by retinal eccentricity, at least in the observed range between 3 to 7.5 deg.

In some studies on the related concept of informational persistence, where letters were used as stimuli, retinal eccentricity and stimulus size were deliberately confounded [33,38] to keep letter legibility constant, which suggests that eccentricity effects should be studied by proportionally adjusting stimulus size. Here we show that, (i) regarding visible persistence, the effects of stimulus size appear to be independent of eccentricity, and (ii) eccentricity by itself did not have any effects. Interestingly, Long and colleagues report the absence of any size effects (confounded with eccentricity effects) on informational persistence under photopic viewing conditions [33,38] using a partial report task and luminance or wavelength defined stimuli. This suggests that informational and visible persistence differ regarding their dependence on stimulus size and thus seem to involve different underlying mechanism. As visible persistence refers to the persistence of a percept, whereas informational persistence [3] refers to abstract stimulus information we find it plausible that spatial parameters should affect the former, but not the latter.

We expected visible persistence to negatively correlate with eccentricity, as mediated by the retinal distribution of P-cells, which would have explained the annulus-size effect of Experiment I as well. Nevertheless, eccentricity did not show any effect in Experiment III, which raises two questions: Can the assumption of P-cell contribution be upheld? How can the results of Experiment I be reconciled with a positive size effect but without an eccentricity effect? As these questions bear implications for both experiments, we deal with them in the General Discussion, suggesting an explanation that unifies the results of Experiment I and III without assuming eccentricity effects.

## General Discussion

Experiments I and III showed that the duration of fading percepts, triggered by transient shapes, depends systematically on spatial parameters. Small annuli persist longer than large annuli, thick annuli persist longer than thin annuli, and large disks persist longer than small disks, irrespective of retinal eccentricity.

Negative effects of annulus size in Experiment I and positive effects of disk size on visible persistence duration in Experiment III seem to contradict each other. The effects of annulus thickness and disk size, however, pointed in the same direction. The latter finding seems to imply that visible persistence duration is a function of stimulus energy. However, Experiment I ruled out such an explanation by showing that small/thick annuli and large/thin annuli differed most in visible persistence despite equal stimulus energy. Additionally, Experiment III showed that visible persistence increases linearly with disk diameter, but not with surface area.

A hypothetical mechanism that unifies these results may behave similarly as *brightness filling-in* [26–28]. Paradiso and Nakayama’s classic study showed that the surface of a uniform bright disk is not represented instantaneously, but is generated by a diffusive spread of brightness information, which starts at the stimulus edges and proceeds inward in a centripetal manner. According to Arrington’s [39] computational model of filling-in, the strength of the contour determines the speed of filling-in. The time course of brightness filling-in, however, is obviously incompatible with the present results on visible persistence duration: Paradiso and Nakayama estimated brightness to spread with a speed of about 110–150 deg/s, whereas visible persistence of transient shapes increased by about 70 ms on average with every degree in diameter, resulting in a speed estimate of about 7 deg/s (degree here refers to the radius, as in Paradiso and Nakayama’s study). Since our stimuli are not defined by brightness, the question is what kind of information is supposed to be filled in. In terms of the taxonomy for perceptual completion phenomena in static images [40], we might describe fading percepts of transient shapes as *modal* completions over time, although *extension* fits the situation better than *completion*, because the latter implies some limit up to which the percept is prolonged, whereas *modal extension* implies no such limit.

To test the filling-in interpretation of our results we scrutinized the parameters of our stimuli that are most crucial for filling-in models, i.e. the strength of the edge signal and the distance to be filled-in [26,27,39]. Filling-in studies typically use radial stimuli, e.g. disks of light, for which filling-in is likely to resemble a centripetal diffusive spread that starts at the outer stimulus edge. Thus, the radius equals the distance to be filled-in. The strength of the edge signal is typically defined by luminance contrast [27,39]. This parameter cannot be directly applied to the transient shapes that we used, as at any given moment there is no mean brightness or texture gradient between stimulus and background. However, there are spatio-temporal differences, defined by changing pixels. The edge signal therefore is likely to scale with the number of flipping pixels at the transient shape’s edge.

Based on these tentative assumptions we tried to linearly predict *VP* in Experiment I from the lengths of the stimulus circumference and the filling-in distance. Fitting a multiple regression of the form *VP* = *b*_0_ + *b*_1_ *C* + *b*_2_ *D*, where C is the summed length of the inner and outer circumference and D the annulus thickness (in pixels) yielded *b*_*0*_ = 534.08, *b*_*1*_ = -0.047, and *b*_*2*_ = 1.626, r = 0.993, p < .01. This suggests that contour strength (defined as contour length) has negative effects (*b*_*1*_) on *VP* because it increases the filling-in speed, whereas annulus thickness has positive effects (*b*_*2*_) on *VP* because it increases the distance to be filled-in.

We then used these estimated regression weights to predict *VP* observed in Experiment III, i.e. the four cell means corresponding to the different disk sizes, averaged across different levels of eccentricity. This prediction was highly successful, as indicated by a correlation between observed and predicted *VP* of r = 0.993, p < .01. We conclude that the stimulus variables *contour length* and *filling-in distance* are sufficient for an accurate quantitative account of visible persistence duration in Experiment I, and, moreover, allow to closely link the findings of Experiment I and III which, on a first look, seemed to contradict each other. It is not too difficult to gain more insight on why this is so: (i) thin annuli persist less than thick annuli because of the shorter travelling distance of the filling-in process. (ii) Large annuli persist less than small annuli because of their longer contours. Finally, (iii) disks increase in visible persistence with increasing radius because increasing the travelling distance more than overcomes the negative effect of increasing contour length.

Such a link between visible persistence and contour processing has been proposed before by Francis and colleagues [9,41,42], in a computational model henceforth called the *boundary erosion model*. In this model, visible persistence corresponds to the erosion of boundary signals in the so-called *boundary contour system* (BCS). The BCS consists of a hierarchical organization of unoriented center-surround cells, simple, complex and hypercomplex cells. These layers interact via inhibitory and excitatory feedback loops to establish boundary segmentation. Persistence is the results of positive feedback interactions that (i) choose a coherent boundary segmentation from among many possible groupings, and (ii) inhibit potential groupings that are weaker [9].

Filling-in, which we assume is related to visible persistence, also plays a major role in the boundary erosion model [41] but there it is claimed not to be directly related to visible persistence. Instead, filling-in is modeled as a process hosted in the so-called *feature contour system* (FCS). Activity in the FCS corresponds to the subject’s perception of a stimulus; however, it is not causally related to visible persistence. As the FCS depends on BCS input, its activity also fades as the BCS activation decays. Our results suggest that both the boundary and the feature contour systems contribute to visual persistence individually.

Furthermore, the model’s application to results on visible persistence explains why visible persistence generated by second-order transients lasts much longer than visible persistence generated by briefly flashed first-order stimuli: the offset of a contour resets the boundary segmentation process by actively suppressing persisting signals. Meyer and Ming [43] have shown that illusory contours generated by a Kanizsa stimulus persisted for up to about 350 ms whereas as real contours persisted for about 200 ms. Francis and Grossberg [41] showed that their model accounted for this effect because the offset of a Kanizsa stimulus did not generate persistence-limiting offset signals at the location of the illusory contour. Some offset signals were generated at the illusion-inducing edges and spread from there thus causing some suppression of visible persistence at stimulus offset. Second-order transients can therefore be expected to produce even longer visible persistence because of the total absence of any offset signals that could limit visible persistence.

The same rationale holds also for *form-from-motion* stimuli [14–18]. Given the large difference in visible persistence between luminance-defined stimuli and form-from-motion stimuli, Wong et al. [18] have concluded that the two types of visible persistence are qualitatively different, with the former corresponding to “low-level sensory representations of iconic visual memory” and the latter corresponding to “object persistence”, which is necessary for binding object elements. In contrast, we conclude that the reason for visible persistence estimates being about ten times shorter than those obtained in classical visible persistence experiments is the result of a visible persistence-limiting offset signal. This signal is neither contained in the form-from-motion stimulus nor in the second-order transients that we employed, as they consist of an isolated transient onset signal, but no sustained signal and no transient offset signal. In line with the boundary erosion model [9,41,42] we therefore propose that in both cases visible persistence is related to a binding process.

## Conclusion

Here we have shown that second order-transients can lead to visible persistence that may last for several tens of a second. Varying the size of the stimuli had systematic, non-trivial effects: thicker annuli persist longer than thin annuli; larger disks persist longer than smaller disks. Although small-thick annuli had the same surface area as large-thin ones, their persistence was maximally different, indicating that surface area alone cannot account for variations in visible persistence. No effects of eccentricity on visible persistence were found. This challenges the temporal integration model which attributes visible persistence solely to the output of sustained visual channels, as the proportion of recruited sustained and transient channels changes dramatically with increasing eccentricity. The observed decrease in visible persistence with increasing annulus radius (keeping thickness constant) is therefore unlikely to be caused by greater eccentricity. Instead, we propose that it may be caused by the increase in total boundary length which in turn is assumed to speed up boundary segmentation, thus decreasing visible persistence. Whether the boundary erosion model actually supports this negative association between contour length and speed of boundary segmentation is beyond the scope of the present article. We claim that future models should take contour length into account and should furthermore test the involvement of filling-in processes in the generation of visible persistence.

## Supporting Information

S1 Fig Animated example of a transient shape. The demonstration shows a transient shape together with a visual reference stimulus. The example shows the large, thick annulus that was used in Experiment I. In the actual experiment, the stimulus sequence would repeat itself just like in this demonstration but a new random dot matrix would be generated for every sequence. (To make sure that the animation works correctly open the figure in a web browser).
(GIF)

S2 Fig Animated example of the proxy stimulus used in Experiment II. The demonstration shows a gray disk embedded in a random dot matrix. The transparency was modulated over time according to the impulse response function described in Method section of Experiment II. Modulating the transparency is technically identical to changing the luminance contrast of the bright and dark pixels in the target region. The average brightness of the target region was always identical to that of the surrounding part of the matrix. (To make sure that the animation works correctly open the figure in a web browser).
(GIF)
