# Supplementary figures and images for: Visible Persistence of Single-Transient Random Dot Patterns: Spatial Parameters Affect the Duration of Fading Percepts
Source: PLoS One. 2015 Sep 8;10(9):e0137091. doi: 10.1371/journal.pone.0137091 (PMC4562503; doi:10.1371/journal.pone.0137091)

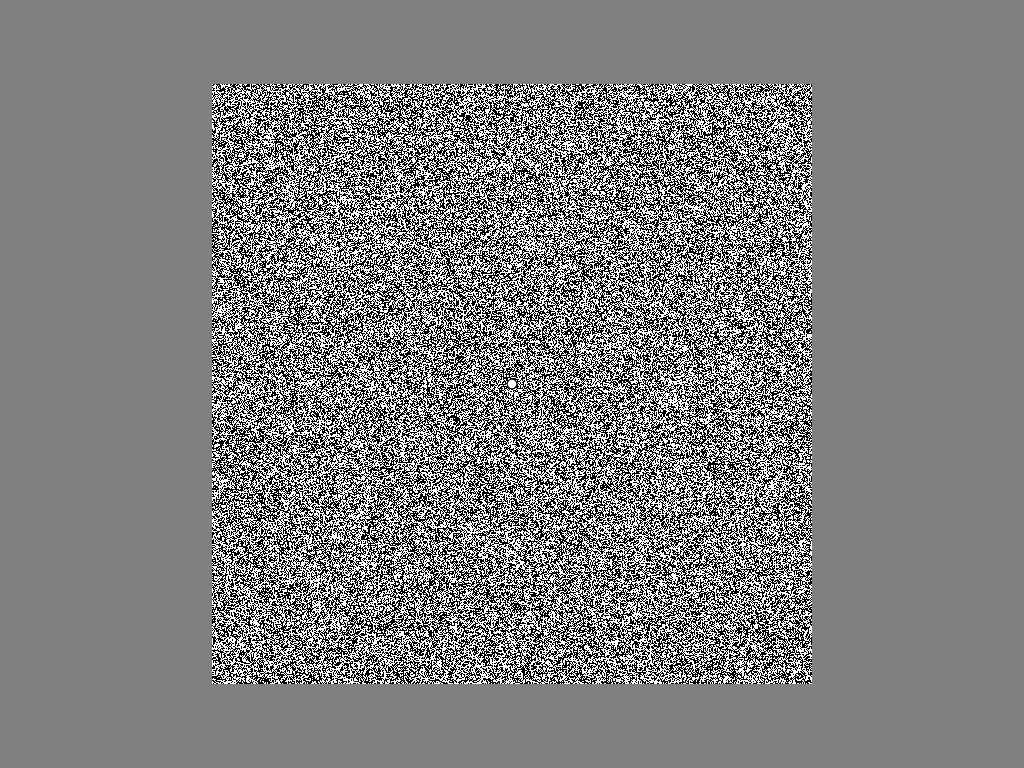

Supplement: S1 Fig — The demonstration shows a transient shape together with a visual reference stimulus. The example shows the large, thick annulus that was used in Experiment I. In the actual experiment, the stimulus sequence would repeat itself just like in this demonstration but a new random dot matrix would be generated for every sequence. (To make sure that the animation works correctly open the figure in a web browser). (GIF) [file pone.0137091.s001.gif]

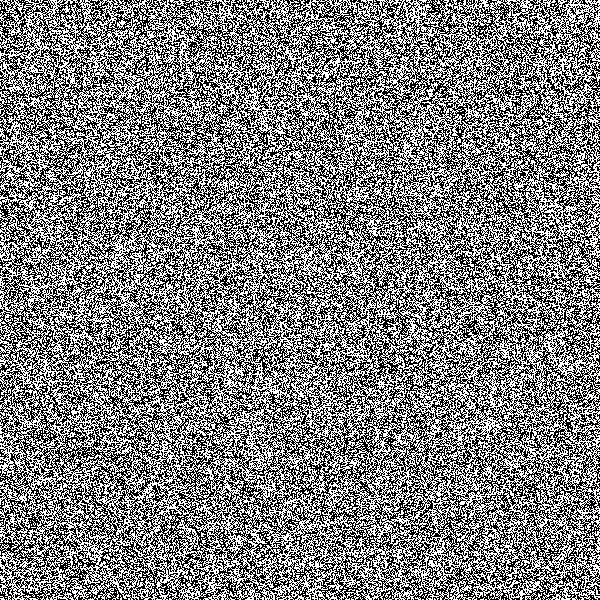

Supplement: S2 Fig — The demonstration shows a gray disk embedded in a random dot matrix. The transparency was modulated over time according to the impulse response function described in Method section of Experiment II. Modulating the transparency is technically identical to changing the luminance contrast of the bright and dark pixels in the target region. The average brightness of the target region was always identical to that of the surrounding part of the matrix. (To make sure that the animation works correctly open the figure in a web browser). (GIF) [file pone.0137091.s002.gif]
